# Sex differences in kinematic adaptations to muscle fatigue induced by repetitive upper limb movements

**DOI:** 10.1186/s13293-018-0175-9

**Published:** 2018-04-19

**Authors:** Jason Bouffard, Chen Yang, Mickael Begon, Julie Côté

**Affiliations:** 1 Department of Kinesiology and Physical Education, McGill University, Montreal, H2W 1S4 Qc Canada; 2 Occupational Biomechanics and Ergonomics Laboratory, Michael Feil and Ted Oberfeld/CRIR Research Centre, Jewish Rehabilitation Hospital, Laval, H7V 1R2 Qc Canada; 3 Département de kinésiologie, Université de Montréal, Laval, H7N 0A5 Qc Canada

**Keywords:** Fatigue, Sex differences, Kinematics, Adaptation, Upper limb, Shoulder, Gender, Musculoskeletal disorders

## Abstract

**Background:**

Muscle fatigue induced by repetitive movements contributes to the development of musculoskeletal disorders. Men and women respond differently to muscle fatigue during isometric single-joint efforts, but sex differences during dynamic multi-joint tasks have not been clearly identified. Moreover, most studies comparing men and women during fatigue development assessed endurance time. However, none evaluated sex differences in kinematic adaptations to fatigue during multi-joint dynamic tasks. The objective of the study was to compare how men and women adapt their upper body kinematics during a fatiguing repetitive pointing task.

**Methods:**

Forty men and 41 women performed repetitive pointing movements (one per second) between two targets while maintaining their elbow elevated at shoulder height. The task ended when participants rated a perceived level of fatigue of 8/10. Trunk, humerothoracic, and elbow angles were compared between the first and last 30 s of the experiment and between men and women. Linear positions of the index finger (distance from the target) and the elbow (arm elevation) as well as movement timing were documented as task performance measures.

**Results:**

Men (7.4 ± 3.2 min) and women (8.3 ± 4.5 min) performed the repetitive pointing task for a similar duration. For both sex groups, trunk range of motion increased with fatigue while shoulder’s and elbow’s decreased. Moreover, participants modified their trunk posture to compensate for the decreased humerothoracic elevation. Movements at all joints also became more variable with fatigue. However, of the 24 joint angle variables assessed, only two *Sex × Fatigue* interactions were observed. Although average humerothoracic elevation angle decreased in both subgroups, this decrease was greater in men (standardized response mean [SRM] − 1.63) than in women (SRM − 1.44). Moreover, the movement-to-movement variability of humerothoracic elevation angle increased only in women (SRM 0.42).

**Conclusion:**

Despite many similarities between men’s and women’s response to fatigue induced by repetitive pointing movements, some sex differences were observed. Those subtle differences may indicate that men’s shoulder muscles were more fatigued than women’s despite a similar level of perceived exertion. They may also indicate that men and women do not adapt the exact same way to a similar fatigue.

**Electronic supplementary material:**

The online version of this article (10.1186/s13293-018-0175-9) contains supplementary material, which is available to authorized users.

## Background

Individuals working in highly constrained and repetitive jobs have more than twice the risk of being diagnosed with a neck and/or shoulder musculoskeletal disorder (neck/shoulder MSD) than those with other types of employment [1]. This high neck/shoulder MSD prevalence is observed even in light but repetitive jobs such as assembly and sorting, requiring muscle activity generally lower than 20% of maximal exertion [1, 2]. Being a woman is another important risk factor for developing work-related neck/shoulder MSD [3, 4]. In a recent meta-analysis, Nordander et al. [2] showed that such gender differences in neck/shoulder MSD exist even when the exposure to mechanical and psychosocial risk factors are equivalent for men and women. This suggests that the women’s higher prevalence of neck/shoulder MSD could at least be partly attributable to sex differences in the internal dose and response associated to the exposure to those same risk factors [5, 6].

Many authors suggested that fatigue may be a precursor to MSD development [6–8]. Fatigue is defined by Enoka and Duchateau as “a disabling symptom in which physical and cognitive function is limited by interactions between performance fatigability and perceived fatigability” [9]. Performance fatigability refers to “the decline in an objective measure of performance over a discrete amount of time” associated with fatigue while perceived fatigability refers to the “changes in sensations that regulate the integrity of the performer.” In turn, changes in overall performance, such as decreased endurance time, can be used as common measures of global fatigability. Indeed, many conditions leading to fatigue such as movement repetitions and maintenance of static non-neutral postures are also known risk factors for work-related MSD [10] and can lead to structural damage because of blood flow occlusion and metabolite accumulation [11]. Interestingly, many studies showed that women are able to produce a constant level of force (i.e., isometric contraction) relative to their maximal force for a longer duration than men (reviewed in [12]). Differences in fatigability were even observed when comparing strength-matched men and women performing intermittent isometric elbow contractions [13]. This apparent advantage for women in muscle endurance during isometric contraction appears in contradiction to their higher risk of MSD development. However, studies that assessed sex differences in fatigue development during dynamic contractions, which are more closely related to muscle actions performed in daily activities, showed much more overlap in men’s and women’s endurance than with isometric contractions [14]. It has been hypothesized that motor adaptations to muscle fatigue could mitigate the MSD risk [7, 15]. Indeed, many redundant structures (e.g., joints, muscles, motor units) compose the motor system and can be controlled in an infinite number of ways to successfully achieve a motor task, a phenomenon referred to as motor abundance [16, 17]. The use of motor abundance can help to prevent fatigue and MSDs by distributing the loads across multiple structures when performing a repetitive task instead of overloading some [15, 18]. Many studies have shown fatigue-related adaptations in the activity of a single muscle or a set of synergistic muscles during highly constrained single-joint tasks [19, 20].

During multi-joint tasks, adaptations in muscle activity [21–23] and joint kinematics [24–27] were also observed. For instance, during a repetitive pointing task (RPT) fatiguing mostly shoulder muscles, observed changes in trunk posture have been thought to compensate for the decline in arm elevation angle to maintain the postural requirement of the task [24]. In addition, most of the kinematic parameters assessed in those studies became more variable from one movement to the other with fatigue development [28, 29]. This increase in movement-to-movement variability is thought to allow the redistribution of mechanical stresses across various structures in response to fatigue [18]. Despite these fatigue-related changes, most of these studies showed that people were able to maintain some general task objectives (e.g., maintain a certain movement frequency, endpoint trajectory) constant, suggesting an ability of the system to reorganize and take advantage of its motor abundance when performing complex movements under challenging conditions such as fatigue.

While the studies cited above showed the complexity of motor adaptations to fatigue, very few compared them between men and women. Srinivasan et al. [22] recently showed that women increased their biceps activity amplitude variability with fatigue during the RPT, while the variability of this muscle decreased in men [22]. Conversely, while trapezius variability increased with fatigue in both sexes, the increase was larger in men than in women [22]. To our knowledge, no studies assessed sex differences in kinematic adaptations to muscle fatigue during a multi-joint task such as the RPT.

The primary objective of the present study was to assess sex differences in fatigue-induced kinematic changes during the RPT. Based on previous electromyography (EMG) findings [22], we expected that only women would increase their elbow movement variability. Moreover, while we expected an increase in shoulder movement variability with fatigue for both sexes as previously observed [28, 29], this change would be greater in men than in women [22]. As the changes in EMG mean amplitude with fatigue have previously been shown to be similar between sexes during the RPT [22], the decrease in elbow and shoulder contribution to the task should not be different between men and women. As a secondary objective, we sought to examine if some Non-Fatigue motor behavior was related to men’s and women’s endurance. Based on the previous finding, we expected that the amount of movement-to-movement variability at the shoulder joint would positively correlate with endurance time, especially in women [21].

## Methods

Eighty-one participants (41 women and 40 men; Table 1) were recruited using advertisement flyers approved by the ethics committee and that were posted on the bulletin boards of the kinesiology, physical education, and rehabilitation departments, and of the research center. The exclusion criteria were any history of mechanical upper limb and/or back pain or injury, neurological, vestibular, or other conditions affecting balance. The protocol was approved by the ethics committee of the Center for Interdisciplinary Research in Rehabilitation of Greater Montreal. Part of the data has been presented in published articles [24, 29, 30] but was never previously analyzed for sex differences.

**Table 1:** Demographic data

	Women (*n* = 41)	Men (*n* = 40)	*p* values*
Age	27.5 ± 8.3 years	29.8 ± 11.4 years	0.736
Height	165.7 ± 6.4 cm	177.2 ± 7.1 cm	< 0.001
Weight	61.1 ± 8.3 kg	73.8 ± 6.1 kg	< 0.001

*Independent samples *t* test, *p* < 0.05

### Protocol

Participants performed the RPT, as first described in Fuller et al. [24]. Briefly, participants repetitively moved their dominant arm between a proximal (30% of arm length) and a distal (100% of arm length) target aligned with their body midline at shoulder height while standing. They maintained a rhythm of one movement per second (2 s for a cycle) set by a metronome. The touch-sensitive cylindrical targets (length 6 cm, radius 0.5 cm Quantum Research Group Ltd) provided auditory feedback when activated to help participants maintain the metronome pace. An elliptically shaped mesh barrier was positioned under participants’ elbow motion trajectory, 10 cm below the target’s midpoint, to ensure it remained elevated during the whole task. Participants rated their level of perceived exertion (RPE) using a Borg CR10 scale every minute [31]. They performed the task until they reached a RPE of 8/10. Participants were unaware of this task termination criterion. Previous studies studying the RPT showed objective signs of performance fatigability such as a decrease of maximal isometric shoulder elevation force and pushing-pulling power generation capacity using the same stopping criterion [24, 32]. EMG signs of fatigue were previously reported in several shoulder and elbow muscles [21, 22].

### Kinematic data acquisition and analysis

Upper body and trunk kinematic data were acquired at 120 Hz using a six-camera motion capture system (MX3 Vicon, Oxford Metrics Ltd., Oxford, UK). Reflective markers (10 mm diameter) were placed on the trunk (C7, T10, manubrium), upper arm (acromioclavicular joint, deltoid insertion, lateral epicondyle), forearm (middle of the forearm, medial and lateral styloid processes), and hand (second metacarpophalangeal joint, index fingertip) [24, 33]. Before performing the RPT, each participant maintained a static position (standing with the arm in anatomical position or elevated at 90° in abduction) to define the kinematic model. Marker trajectories were low-pass filtered at 15 Hz (zero lag, Butterworth, fourth order).

**Fig. 1:**
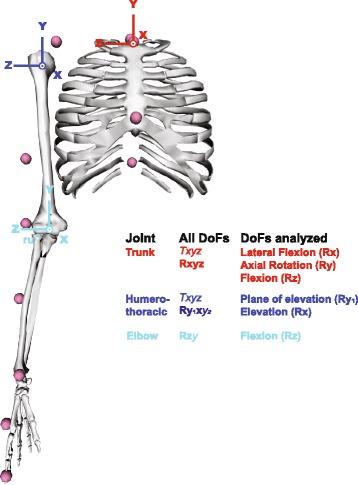
Model used to analyze kinematic data. Joint coordinate systems are presented for the trunk-global, humerothoracic, and elbow joints. Wrist and hand joints are not presented here for clarity. The transformation orders (degrees of freedom, DoF) are presented for each joint. Transformations written in bold characters and presented with their name in the table represent the degrees of freedom reported in the “Results” section. Note that elbow pronation-supination (Ry) occurs at the radioulnar (ru) joint. T, translation; R, rotation

A generic kinematic model adapted from the Standford VA model available in OpenSim [34, 35] was scaled using static trial data. The model included the following degrees-of-freedom: trunk-global (three translations [XYZ], three rotations [XYZ]), humerothoracic (three translations [XYZ], three rotations [Y_1_XY_2_]), and elbow [ZY] as well as multiple joints at the wrist and hand (Fig. 1). Trunk lateral flexion (X rotation), axial rotation (Y rotation), and flexion (Z rotation), and humerothoracic plane of elevation (Y_1_ rotation), and elevation (X rotation) as well as elbow flexion (Z rotation) waveforms were reported for analysis [36]. As in Wu et al. [36], the humerothoracic elevation angle is the angle between longitudinal axes of the humerus and trunk. The plane of elevation is the plane in which this elevation is observed: 0° is abduction and 90° is flexion. Dominant index (IDX) and lateral epicondyle (ELB) marker positions were also analyzed, as they are directly relevant to the task goals, pointing toward the target for the former and maintaining the elbow above the mesh for the latter. All data were then partitioned into individual forward and backward pointing movements using the target switch signal or peak IDX antero-posterior velocity. For each movement, the following variables were computed:

- Each joint’s average angle (to represent average posture) and range of motion (to represent movement amplitude)
- The *Euclidean* distance between IDX marker and the distal target’s center at the end of the movement (movement error)
- The average *vertical* distance between ELB marker and the mesh barrier (elbow height)
- Movement duration timing error (|movement duration – 1 s|).

The mean value and movement-to-movement variability (standard deviation) of each joint angular variable (range of motion and average angle) and of movement errors were calculated across all movements for one condition (Non-Fatigue: first 30 s or Fatigue Terminal: last 30 s). Only mean values were calculated for the elbow height, movement duration, and timing error variables. Only data from forward movements are reported here.

### Statistical analysis

Men’s and women’s age, height, weight, and endurance time were compared with independent sample *t* tests. Two-way ANOVAs (sex [between subjects: men vs women] × Fatigue [repeated measures: Non-Fatigue vs Fatigue Terminal]) were performed on each of the variables. Post hoc paired or independent sample *t* tests with Holm corrections [37] were performed when interactions were observed for the following planned comparisons: women Non-Fatigue vs women Fatigue Terminal, men Non-Fatigue vs men Fatigue Terminal, women Non-Fatigue vs men Non-Fatigue, and women Fatigue Terminal vs men Fatigue Terminal. The standardized response mean (SRM) was calculated for each variable for men and women with its confidence interval to judge the similarities and differences in motor adaptations to muscle fatigue [38]. The SRM was interpreted qualitatively according to Cohen’s standards as low (0.2 < SRM < 0.5), medium (0.5 < SRM < 0.8), or high (SRM > 0.8) [39].

To assess the association between each Non-Fatigue joint angle variable and endurance time, Pearson correlation coefficients with their 95% confidence interval were computed.

## Results

Sex subgroups did not differ in terms of age (*t* test, *p* = 0.736), but men were taller (*p* < 0.001) and heavier (*p* < 0.001) than women (Table 1). Endurance time during the RPT was not different between women (8.3 ± 4.5 min) and men (7.4 ± 3.2 min) (*p* = 0.96).

### Effects of movement repetitions and sex on motor performance

**Fig. 2:**
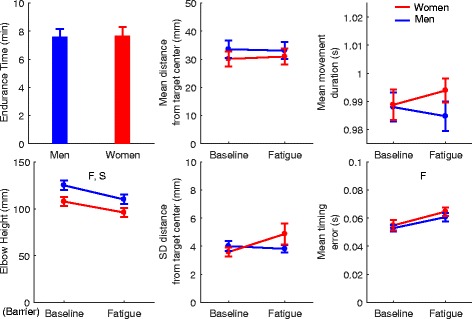
Effects of Sex and Fatigue on task performance variables. F (main effect of Fatigue), S (main effect of Sex), S×F (Sex × Fatigue interaction)

Most of the RPT performance variables did not change with fatigue in men or in women (Fig. 2). Indeed, no Fatigue, Sex, or Sex × Fatigue effects were observed for the movement error, movement error variability, and movement duration variables (all *F* < 3.195, *p* > 0.078). However, elbow height decreased with fatigue (main effect of Fatigue: *F*_1,79_ = 45.019, *p* < 0.001). Moreover, men’s elbow was significantly higher relative to the mesh barrier when compared to women (main effect of sex: *F*_1,79_ = 5.412, *p* = 0.023). Importantly, at the end of the experiment, elbow height was still at least 10 mm over the mesh barrier (whole sample minimum), with an average elbow height of 96 ± 32 mm for women and 110 ± 32 mm for men. There was also a small but consistent increase in movement timing error with fatigue in both sex subgroups (main effect of Fatigue: *F*_1,79_ = 10.833, *p* = 0.001).

### Effects of movement repetitions and sex on upper body mean kinematic parameters

**Fig. 3:**
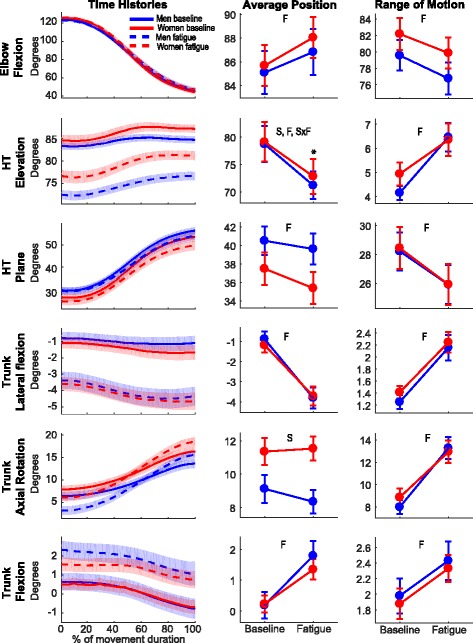
Effects of Sex and Fatigue on mean kinematic behavior. *Left panel*—men’s (blue) and women’s (red) mean joint angle time histories during Non-Fatigue (full lines) and Fatigue Terminal (dashed lines) movements. *Center panel*—mean average angle for each DoF. *Right panel*—mean range of motion for each DoF. F (main effect of Fatigue), S (main effect of Sex), S×F (Sex × Fatigue interaction). *Post hoc analysis showing differences between men and women. HT, humerothoracic

Non-Fatigue waveforms of the different joint angles (Fig. 3, left panels) show that elbow flexion and humerothoracic plane of elevation contributed importantly to the dynamic component of the reaching task because of their broad ranges of motion. Moreover, the large deviation from the anatomical position and the low range of motion observed for the humerothoracic elevation degrees of freedom (DoF) illustrate the important postural demands of the task at this joint. All trunk angles had a minimal contribution to the task before fatigue (low range of motion and angular position close to neutral). However, trunk contribution to the task increased with fatigue, while elbow and shoulder contributions decreased. This multi-joint movement reorganization with fatigue is highlighted by main effects of Fatigue for all joint angle variables (mean values) except for the average trunk axial rotation angle (Table 2).

**Table 2:** ANOVAs for joint kinematic variables

		Mean values			Movement-to-movement variability (SD)		
		Fatigue	Sex	Sex × Fatigue	Fatigue	Sex	Sex × Fatigue
Elbow flexion	Range of motion	*F*_*1,79*_ *= 8.82*	*F*_1,79_ = 1.44	*F*_1,79_ = 0.17	*F*_*1,79*_ *= 22.77*	*F*_*1,79*_ *= 5.75*	*F*_1,79_ = 0.70
		*p = 0.004*	*p* = 0.234	*p* = 0.678	*p < 0.001*	*p = 0.019*	*p* = 0.407
	Average angle	*F*_*1,79*_ *= 8.27*	*F*_1,79_ = 0.11	*F*_1,79_ = 0.57	*F*_1,79_ = 3.06	*F*_*1,79*_ *= 11.39*	*F*_1,79_ = 0.19
		*p = 0.005*	*p* = 0.741	*p* = 0.451	*p* = 0.195	*p = 0.001*	*p* = 0.662
HT plane	Range of motion	*F*_*1,79*_ *= 6.70*	*F*_1,79_ = 0.01	*F*_1,79_ < 0.01	*F*_*1,79*_ *= 24.65*	*F*_1,79_ = 2.69	*F*_11,79_ = 0.38
		*p = 0.011*	*p* = 0.910	*p* = 0.986	*p < 0.001*	*p* = 0.105	*p* = 0.537
	Average angle	*F*_*1,79*_ *= 8.57*	*F*_1,79_ = 2.98	*F*_1,79_ = 0.64	*F*_*1,79*_ *= 9.42*	*F*_1,79_ = 1.22	*F*_1,79_ = 0.49
		*p = 0.004*	*p* = 0.088	*p* = 0.428	*p = 0.003*	*p* = 0.274	*p* = 0.484
HT elevation	Range of motion	*F*_*1,79*_ *= 25.89*	*F*_1,79_ = 0.84	*F*_1,79_ = 0.96	*F*_*1,79*_ *= 21.39*	*F*_1,79_ = 0.70	*F*_1,79_ = 0.50
		*p < 0.001*	*p* = 0.362	*p* = 0.331	*p < 0.001*	*p* = 0.404	*p* = 0.482
	Average angle	*F*_*1,79*_ *= 191.55*	*F*_*1,79*_ *= 7.05*	*F*_*1,79*_ *= 4.77*	*F*_*1,79*_ *= 5.36*	*F*_*1,79*_ *= 7.17*	*F*_*1,79*_ *= 4.09*
		*p < 0.001*	*p = 0.010*	*p = 0.032*	*p = 0.023*	*p = 0.009*	*p = 0.047*
Trunk lateral flexion	Range of motion	*F*_*1,79*_ *= 55.56*	*F*_1,79_ = 0.48	*F*_1,79_ = 0.10	*F*_*1,79*_ *= 51.01*	*F*_1,79_ = 1.31	*F*_1,79_ = 1.07
		*p < 0.001*	*p* = 0.489	*p* = 0.757	*p < 0.001*	*p* = 0.256	*p* = 0.305
	Average angle	*F*_*1,79*_ *= 108.36*	*F*_1,79_ = 0.27	*F*_1,79_ = 0.81	*F*_*1,79*_ *= 47.25*	*F*_1,79_ = 0.01	*F*_1,79_ = 0.04
		*p < 0.001*	*p* = 0.602	*p* = 0.128	*p < 0.001*	*p* = 0.944	*p* = 0.264
Trunk axial rotation	Range of motion	*F*_*1,79*_ *= 87.18*	*F*_1,79_ = 0.31	*F*_1,79_ = 1.58	*F*_*1,79*_ *= 34.04*	*F*_1,79_ = 0.13	*F*_1,79_ = 0.09
		*p < 0.001*	*p* = 0.581	*p* = 0.213	*p < 0.001*	*p* = 0.719	*p* = 0.765
	Average angle	*F*_1,79_ = 0.53	*F*_*1,79*_ *= 6.34*	*F*_1,79_ = 0.61	*F*_*1,79*_ *= 26.55*	*F*_1,79_ = 1.58	*F*_1,79_ = 0.28
		*p* = 0.470	*p = 0.014*	*p* = 0.437	*p < 0.001*	*p* = 0.212	*p* = 0.602
Trunk flexion	Range of motion	*F*_*1,79*_ *= 13.92*	*F*_1,79_ = 0.10	*F*_1,79_ < 0.01	*F*_*1,78*_ *= 33.97*	*F*_1,78_ = 0.03	*F*_1,78_ = 0.02
		*p < 0.001*	*p* = 0.753	*p* = 0.953	*p < 0.001*	*p* = 0.872	*p* = 0.878
	Average angle	*F*_*1,79*_ *= 53.25*	*F*_1,79_ = 0.24	*F*_1,79_ = 1.61	*F*_*1,78*_ *= 42.78*	*F*_1,78_ = 1.18	*F*_1,78_ = 0.38
		*p < 0.001*	*p* = 0.627	*p* = 0.208	*p < 0.001*	*p* = 0.281	*p* = 0.539

*F* and *p* values are presented for each two-way ANOVA (Sex [between subjects: women vs men] × Fatigue [repeated measures: Non-Fatigue vs Fatigue Terminal]). Itatic fonts present statistically significant effects (*p*<0.05)

*HT* humerothoracic

In addition to the various common fatigue-related kinematic changes between men and women, there was a Sex × Fatigue interaction for the average humerothoracic elevation angle. Post hoc analyses showed that while humerothoracic elevation decreased with fatigue for both men (− 9.8° ± 6.0°; *t*_39_ = 10.305, *p*_corrected_ < 0.001) and women (− 7.2° ± 5.0°; *t*_40_ = 9.211, *p*_corrected_ < 0.001), this decrease was more important in men. Indeed, men’s average humerothoracic elevation angle was lower than women’s at the end of the experiment (*t*_79_ = 3.210, *p*_corrected_ = 0.004) while no differences were observed at Non-Fatigue (*t*_79_ = 1.456, *p*_corrected_ = 0.149). Finally, women’s trunk was more rotated (10.9° ± 5.0°) than men’s (8.9° ± 4.4°) during the whole experiment, so that their reaching shoulder was more advanced.

### Effects of muscle fatigue and sex on upper body movement-to-movement variability

**Fig. 4:**
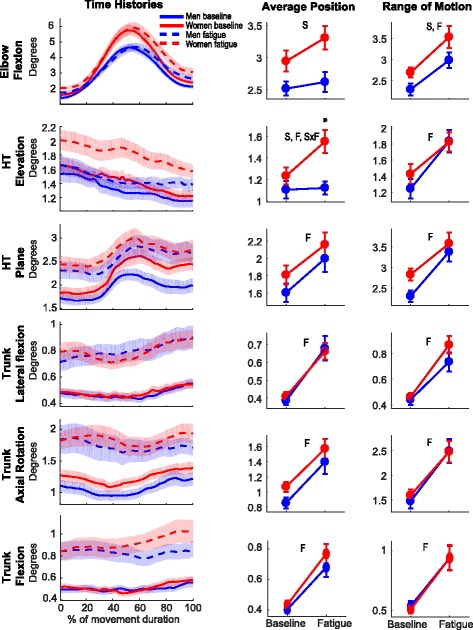
Effects of Sex and Fatigue on kinematic behavior movement-to-movement variability. *Left panel*—men’s (blue) and women’s (red) joint angle standard deviation time histories during non-fatigue (full lines) and Fatigue Terminal (dashed lines) movements. *Center panel*—standard deviation of average angle for each DoF. *Right panel*—standard deviation of the range of motion for each DoF. F (main effect of Fatigue), S (main effect of Sex), S×F (Sex × Fatigue interaction). *Post hoc analysis showing differences between men and women. HT, humerothoracic

Movement-to-movement variability (standard deviation) significantly increased with fatigue for all assessed variables except for the average elbow flexion angle (Table 2, Fig. 4). There was also a Sex × Fatigue interaction for the average humerothoracic elevation angle movement-to-movement variability as women became more variable with fatigue (*t*_40_ = − 2.699, *p*_corrected_ = 0.030), but not men (*t*_39_ = − 0.249, *p*_corrected_ = 0.804). In line with this interaction, average humerothoracic elevation angle standard deviation was greater for women than men once fatigued (*t*_77.4_ = 3.241, *p*_corrected_ = 0.008), despite similar variability at the beginning of the task (*t*_79_ = 1.004, *p*_corrected_ = 0.636). Women’s elbow flexion average angle and range of motion were more variable than men’s both before and after fatigue.

### Responsiveness of kinematics variables to RPT-induced changes in men and women

**Fig. 5:**
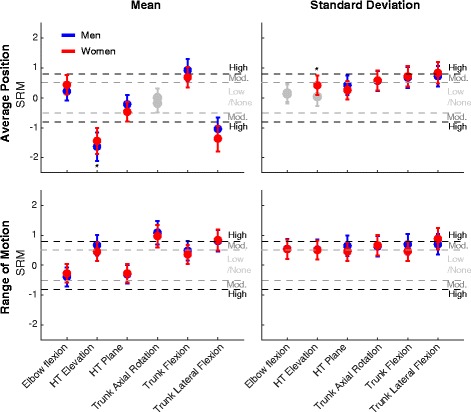
Summary of results. Fatigue-related effect size for men (blue) and women (red) for all computed joint angle variables. Standardized response mean (SRM). Gray symbols indicate non-significant effect. *Sex × Fatigue interactions. HT, humerothoracic

While almost all joint angle variables changed with fatigue (main effects of Fatigue on 22/24 variables), the amplitude of those changes varied (Fig. 5). The most consistent change both in men (SRM [95% CI] = − 1.63 [− 2.11 to − 1.15]) and in women (SRM [95% CI] = − 1.44 [− 1.88 to − 1.00]) was a decrease in average humerothoracic elevation angle. Other variables that were highly responsive to RPT-induced changes (SRM > 0.8) were average trunk lateral flexion angle (mean and movement-to-movement variability [men and women]), trunk lateral flexion range of motion (mean [men and women] and movement-to-movement variability [women]), average trunk flexion (mean [men]), and trunk axial rotation range of motion (mean [men and women]).

### Association between kinematic variables and endurance time

Significant correlations between Non-Fatigue motor behavior and endurance time were observed for only 4 out of 20 possibilities, and correlations were weak (Additional file 1: Table S1). For men, endurance time correlated significantly and negatively with Non-Fatigue mean (*p* = 0.036; *r* [95% CI] = − 0.334 [− 0.025 to − 0.584]) and movement-to-movement variability (*p* = 0.033; *r* [95% CI] = − 0.338 [− 0.023 to − 0.587]) of humerothoracic elevation range of motion. Movement-to-movement variability of shoulder average elevation angle (*p* = 0.032; *r* [95% CI] = − 0.339 [− 0.031 to − 0.589]) was also weakly and negatively related to endurance time in men. For women, only Non-Fatigue variability of trunk flexion (*p* = 0.032; *r* [95% CI] = 0.336 [0.032 to 0.584]) was (positively) associated with the time needed to reach a RPE of 8/10.

## Discussion

This study assessed the effects of sex on upper body kinematic adaptations to muscle fatigue during a repetitive pointing task through a secondary analysis of published [24, 30, 40] and unpublished data. Objective signs of muscle fatigue such as an increase in upper trapezius, anterior deltoid, biceps brachii, and triceps brachii EMG activity have been previously reported [22]. Moreover, a decrease in maximal shoulder elevation force and in maximal pushing and pulling power generation capacity has been observed at the end of the RPT [24, 32]. As previously shown, fatigue induced during the RPT is associated with changes occurring in the whole body [24, 28, 41]. Despite those numerous changes in joint movements, variables describing task performance were only minimally affected by fatigue. Many similarities were observed between men and women in terms of endurance time and fatigue-related kinematic adaptations. However, men and women modified differently their average humerothoracic elevation angle and its movement-to-movement variability with fatigue. Only 4 out of 20 variables describing Non-Fatigue kinematic behavior were associated with endurance time, and correlations were weak.

It is now well documented that upper limb fatigue leads to complex kinematic reorganization involving the whole body [24, 28, 41, 42]. The high number of fatigue-related changes in individual joint angles, ranges of motion, and movement-to-movement variability observed in the present study is in line with previous findings obtained during the same [24, 28] and other tasks [42]. The most consistent change observed in the present study was a decrease in the average humerothoracic elevation angle, which probably indicates the primary effect of fatigue. Similar changes in the arm posture were also observed during a ratcheting task performed after a fatiguing protocol targeting shoulder flexors [42]. Conversely, only subtle changes in arm kinematics were observed during the ratcheting task when fatigue was induced in the forearm muscles [42]. Fatigue induced in the elbow flexors decreased elbow movements during repetitive sawing and hammering tasks, while shoulder movements remained unchanged or increased [25, 26]. Taken together, those studies indicate that kinematic changes related to the primary effect of fatigue are highly task-specific. However, in most of those experiments [25, 42], trunk movements increased with fatigue, as in the present study. The increase in trunk contribution to the task may represent a general strategy used to compensate for upper limb fatigue during multi-joint dynamic tasks.

### Men’s and women’s kinematic changes with fatigue during the repetitive pointing task

Many studies have previously shown that women are more fatigue-resistant when sustaining an isometric contraction or performing series of high-intensity intermittent contractions (reviewed in [12]). However, during dynamic tasks, sex differences in fatigability are less consistent [14, 22]. In the present study, endurance times, based on perceived fatigue, were similar between men and women. Nevertheless, mean humerothoracic elevation angle decreased more in men than in women. This may indicate that humerothoracic elevators reached a higher intensity of fatigue in male participants. It is known that shoulder elevation maximal force decreases by about 5% by the end of the RPT [24]. However, significant inter-individual differences (standard deviation = 170% of the mean reported changes) exist in the amount of muscle force decrease following the RPT. Those inter-individual differences may be critical when studying the effects of individual factors, such as participants’ sex. Hunter et al. [13] showed that women rated greater levels of perceived exertion than men for a similar time point relative to time to task failure during intermittent isometric elbow flexion (e.g., 75% of the time to task failure). Moreover, following fatiguing knee dynamic contractions, men’s maximal force decreased more than women’s despite similar subjective fatigue ratings [43]. In line with those results, it could be argued that men were closer to their actual time to task failure and had a greater force decrease than women at a RPE of 8/10 in our study. This could explain why men’s humerothoracic elevation decreased more than women’s. However, if sex differences in the RPE response curve were the sole factor explaining these results, we would expect Sex × Fatigue interactions on most of the assessed variables as evidence that men would indeed have greater fatigue-related changes than women. However, since sex differences in kinematic adaptations were observed only in a single DoF, it is unlikely that the choice of stoppage criterion explained all our findings.

Another specificity of the present report is the multi-joint dynamic nature of the motor task when compared to the single-joint isometric contractions reported in most of the previous studies comparing men’s and women’s fatigability [12]. Although a significant decrease in maximal force after the RPT was observed in shoulder elevators [24], it is likely that fatigue developed at different sites of the body simultaneously for some participants. For instance, previously observed increases in EMG activity of the trapezius, anterior deltoid, biceps brachii, and triceps brachii suggest some muscle fatigue for both the shoulder and elbow muscles during the RPT [22]. However, men and women differed on some variables such as elbow flexion variability as well as mean trunk rotation from the beginning of the RPT. This indicates that they were not doing exactly the same task, which may influence the structures most at risk of developing muscle fatigue. Moreover, the RPT may lead to a preferential development of muscle fatigue in different structures between sexes. Based on the literature on sex differences in fatigability, we may expect men being more challenged by the postural (isometric) component of the task [12]. Indeed, we observed a greater decrease in humerothoracic elevation, a DoF mostly involved in the postural component of the task, in male participants. Moreover, three variables describing baseline behavior at this DoF out of four were associated with endurance time, but only in men. Men’s greater sensibility to fatigue induced by the postural component of the RPT may be related to their lower concentration of type 1 muscle fibers and lower muscle perfusion [12]. The greater level of muscle fatigue experienced by men in the muscles involved in humerothoracic elevation may also be related to anthropometric differences. Men being heavier and having heavier upper limbs, the absolute force they must produce during the RPT is larger.

### Men’s and women’s variability during the repetitive pointing task

Most kinematic adaptations during the RPT were similar between men and women. However, in addition to the greater decrease in humerothoracic elevation with fatigue for men when compared to women, changes in humerothoracic elevation variability also differed between sexes. It increased with fatigue, but only in women. This suggests that men and women use slightly different kinematic strategies to adapt to muscle fatigue, with men modifying their mean movements to a greater instance and women increasing movement-to-movement variability. By decreasing mean humerothoracic elevation angle, men may reduce the amount of force produced by humerothoracic elevators. On the other hand, women’s strategies may be to increase movement-to-movement variability to spread the load necessary to perform the RPT across redundant structures from one repetition to the other. In their recent study on EMG data, Srinivasan et al. [22] also showed sex differences in motor adaptations during the RPT. Men increased more their upper trapezius muscle EMG variability than women. Moreover, they showed that with fatigue, biceps EMG movement-to-movement variability increased in women but decreased in men. Based on those results, the authors suggested that women used a more elbow-based strategy to maintain their task performance despite fatigue while men’s strategy targeted more the shoulder. We did not find any evidence of the more elbow-based strategy in women from our kinematic data. The relationships between EMG data and movement kinematics are not straightforward, and many methodological [44], physiological [45], and biomechanical [46] factors can alter them. For instance, the biceps brachii, for which different fatigue-related adaptations have been shown between men and women [22], is a bi-articular muscle. Changes in its activation can have an impact at the elbow and/or at the shoulder joint. Nevertheless, the results of both the current study and that of Srinivasan et al. point to the same conclusion that men and women’s fatigue adaptations, although similar, are not exactly identical [21, 22].

## Conclusion

The present study shows many similarities between men and women in terms of endurance time and kinematic adaptations during the RPT. However, men decreased more their humerothoracic elevation than women. Conversely, only women increased humerothoracic elevation movement-to-movement variability. Those subtle sex differences could indicate that men’s humerothoracic elevator muscles were more fatigued than women’s despite a similar perceived exertion or that they adapted differently to a similar level of muscle fatigue. Finally, different pre-fatigue movement parameters are related to endurance time in men vs women. Those results highlight the complexity of the phenomenon of fatigue and its impact on motor behavior. More studies are needed in order to determine if fatigue imposes a sex-specific load on the motor system, which could in turn explain sex-gender differences in mechanisms of MSD.

## Additional file

Additional file 1: Correlation between each kinematic variable Non-Fatigue value and endurance time for men and women. HT: Humerothoracic, p: *p* value, r: Pearson correlation coefficient, rup/rlow: upper and lower bounds of r's 95% confident interval. Significant correlations are indicated in red. (XLSX 12 kb)
